# Wearables for Measuring the Physical Activity and Sedentary Behavior of Patients With Axial Spondyloarthritis: Systematic Review

**DOI:** 10.2196/34734

**Published:** 2022-08-22

**Authors:** Julie Soulard, Thomas Carlin, Johannes Knitza, Nicolas Vuillerme

**Affiliations:** 1 Université Grenoble Alpes, AGEIS La Tronche France; 2 LabCom Telecom4Health Orange Labs & Université Grenoble Alpes CNRS, Inria, Grenoble INP-UGA Grenoble France; 3 Grenoble Alpes University Hospital Grenoble France; 4 Department of Internal Medicine 3–Rheumatology and Immunology Friedrich-Alexander University, Erlangen-Nürnberg, and Universitätsklinikum Erlangen Erlangen Germany; 5 Deutsches Zentrum für Immuntherapie Friedrich-Alexander University Erlangen-Nürnberg and Universitätsklinikum Erlangen Erlangen Germany; 6 Institut Universitaire de France Paris France

**Keywords:** axial spondyloarthritis, rheumatology, physical activity, sedentary behavior, objective measures, wearable, mobile health, mHealth, eHealth, systematic review, mobile phone

## Abstract

**Background:**

Axial spondyloarthritis (axSpA) is an inflammatory rheumatic disease associated with chronic back pain and restricted mobility and physical function. Increasing physical activity is a viable strategy for improving the health and quality of life of patients with axSpA. Thus, quantifying physical activity and sedentary behavior in this population is relevant to clinical outcomes and disease management. However, to the best of our knowledge, no systematic review to date has identified and synthesized the available evidence on the use of wearable devices to objectively measure the physical activity or sedentary behavior of patients with axSpA.

**Objective:**

This study aimed to review the literature on the use of wearable activity trackers as outcome measures for physical activity and sedentary behavior in patients with axSpA.

**Methods:**

PubMed, PEDro, and Cochrane electronic databases were searched in July 2021 for relevant original articles, with no limits on publication dates. Studies were included if they were original articles, targeted adults with a diagnosis of axSpA, and reported wearable device–measured physical activity or sedentary behavior among patients with axSpA. Data regarding the study’s characteristics, the sample description, the methods used for measuring physical activity and sedentary behavior (eg, wearable devices, assessment methods, and outcomes), and the main results of the physical activity and sedentary behavior assessments were extracted.

**Results:**

A total of 31 studies were initially identified; 13 (13/31, 42%) met the inclusion criteria, including 819 patients with axSpA. All the studies used accelerometer-based wearable devices to assess physical activity. Of the 13 studies, 4 (4/31, 31%) studies also reported outcomes related to sedentary behavior. Wearable devices were secured on the wrists (3/13 studies, 23%), lower back (3/13, 23%), right hip (3/13, 23%), waist (2/13, 15%), anterior thigh (1/13, 8%), or right arm (1/13, 8%). The methods for reporting physical activity and sedentary behavior were heterogeneous. Approximately 77% (10/13) of studies had a monitoring period of 1 week, including weekend days.

**Conclusions:**

To date, few studies have used wearable devices to quantify the physical activity and sedentary behavior of patients with axSpA. The methodologies and results were heterogeneous, and none of these studies assessed the psychometric properties of these wearables in this specific population. Further investigation in this direction is needed before using wearable device–measured physical activity and sedentary behavior as outcome measures in intervention studies in patients with axSpA.

**Trial Registration:**

PROSPERO CRD42020182398; https://tinyurl.com/ec22jzkt

**International Registered Report Identifier (IRRID):**

RR2-10.2196/23359

## Introduction

Axial spondyloarthritis (axSpA) is a chronic inflammatory rheumatic disease that can cause inflammatory back pain, structural damage, and disability [1]. New therapeutic agents allow for effective therapy [2]. Physical activity should be an integral part of standard care, according to current guidelines [3], because of its multiple health benefits, including pain reduction [4,5], increased mobility [4-6], physical function [5-8], and cardiorespiratory fitness [9], which ultimately reduces disease activity [4-6,8,10]. Furthermore, increasing physical activity represents a viable strategy for improving quality of life [7,11] and reducing the psychological comorbidities of patients with axSpA [7]. Physical activity is a predictor of mortality and cardiovascular events in the general population [12]. In addition, high-intensity exercises (12 weeks of endurance and strength exercises) have been reported to significantly improve disease activity and reduce cardiovascular risks in patients with axSpA [13]. However, a good therapeutic response depends on short symptom duration and close disease monitoring [14]. A recent review reported that most measures of physical activity used in patients with axSpA were “subjective and limited by patient recall, reporting bias, and relatively short study intervals” [15].

Wearable technology comprises “a device fitted to the participant’s body which detects and collects data” [16]. These wearables can include accelerometers, pedometers, or inertial measurement units, which are small and transportable. They can be advantageously used to monitor physical activity data under real-world conditions in various chronic populations [16-18], including patients with axSpA [11,19-28]. By allowing longitudinal physical activity monitoring, these devices can remotely monitor the disease and evolution of health status in patients with axSpA [20,29]. Indeed, greater disease activity is associated with lower levels of physical activity in axSpA and could help detect flares [20,29].

In addition to measuring physical activity, in a complementary manner, wearables can measure the time spent sitting, also called sedentary behavior. Indeed, it is of particular interest in patients with axSpA as the more time patients with axSpA spend sitting, the greater the association with disease activity [27], decreased physical function [27], and decreased quality of life [30].

Thus, physical activity and sedentary behavior assessments using wearable devices represent an attractive and feasible health monitoring option for patients with axSpA. Interestingly, a multicentric prospective observational study, which involved 157 patients with chronic inflammatory rheumatic diseases, found good acceptability of wearing activity trackers for physical activity assessment in this population [31].

However, to the best of our knowledge, no study has assessed the use of wearables to objectively monitor physical activity and sedentary behavior in patients with axSpA. We designed the present review to identify and synthesize the currently available evidence on the use of wearable activity trackers as outcome measures for physical activity and sedentary behavior in patients with axSpA [32]. We aimed to answer the following research question: which wearable devices, assessment methods, and associated outcomes are commonly used to quantify physical activity or sedentary behavior among patients with axSpA?

## Methods

This systematic review’s protocol was developed based on the PRISMA (Preferred Reporting Items for Systematic Reviews and Meta-Analyses) statement. It was registered in PROSPERO (International Prospective Register of Systematic Reviews; CRD42020182398) and was published in November 2021 [32].

### Inclusion Criteria

Studies were included if they (1) were original articles published in English-language peer-reviewed journals, (2) targeted adults (aged ≥18 years) with a diagnosis of axSpA, and (3) reported wearable device–measured physical activity or sedentary behavior among patients with axSpA.

Studies were excluded if they (1) were case reports, abstracts, editorials, conference abstracts, letters to the editor, reviews, or meta-analyses or (2) did not use wearable devices to quantify the physical activity or sedentary behavior of patients with axSpA.

### Data Sources and Search Strategy

In July 2021, we conducted searches with no date restrictions in 3 electronic bibliographic databases (PubMed, PEDro, and Cochrane). The Boolean operators *AND* and *OR* were used to combine keywords relevant to the population, wearable devices, and the outcomes of physical activity or sedentary behavior and were searched in all fields. The detailed search strategy is presented in the review protocol recently published in *JMIR Research Protocols* [32].

### Study Selection

**Figure 1:**
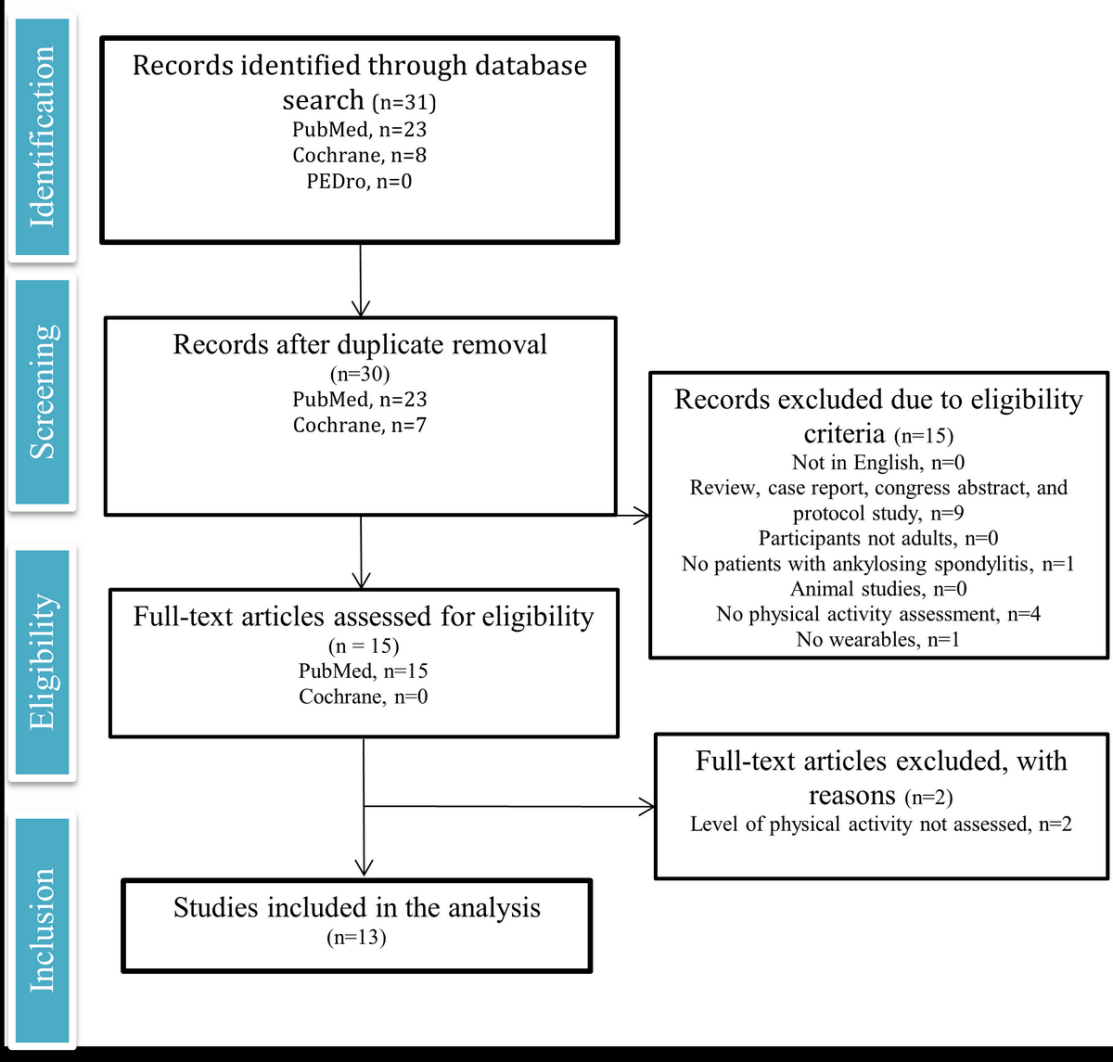
PRISMA (Preferred Reporting Item for Systematic Reviews and Meta-Analyses) flowchart of the selection process.

A total of 2 independent reviewers (TC and JS) screened the titles, abstracts, and keywords of all the studies found in the search to identify potentially relevant articles. Duplicates were manually removed. The selected full-length text articles were then screened for eligibility according to the criteria abovementioned. In cases of disagreement, the reviewers reached a consensus through discussion. If their disagreement persisted, a third reviewer (NV) was asked to make the final decision. In accordance with the PRISMA guidelines [33], a flow chart was constructed to summarize each step of the selection process with its corresponding number of citations (Figure 1).

### Risk of Bias in Individual Studies

As indicated in the published review protocol [32], as the purpose of this review was not to assess the clinical effects of interventions, we did not perform a risk of bias assessment [34-36]. Indeed, as mentioned in the *Introduction* section, this review was designed to identify and synthesize the available evidence on the use of wearable devices to quantify physical activity or sedentary behavior among patients with axSpA.

### Data Extraction

Data extraction was performed independently by 2 reviewers (TC and JS) who were not blinded to the authors or journals. Information was extracted on (1) the study’s characteristics, (2) the sample description, (3) the methods used for measuring physical activity and sedentary behavior (eg, wearable devices, assessment methods, and outcomes), and (4) the main results of the physical activity and sedentary behavior assessments.

### Data Synthesis and Analysis

Owing to the significant heterogeneity of data types, we decided to perform only a narrative synthesis [37,38]. As per the data extraction strategy, the tables and figures found in this review only summarize the available information on wearable devices used to objectively assess the physical activity and sedentary behavior of patients with axSpA.

## Results

### Study Selection

The study selection process is illustrated in Figure 1. A preliminary search of the 3 electronic bibliographic databases identified 31 citations. A duplicate was removed, leaving 97% (30/31) of unique records for preliminary screening, focusing on the title, abstract, and keywords. Finally, of the 30 articles, 13 (43%) met our eligibility criteria and were included in this review.

### Study Characteristics

The characteristics of the studies included (N=13) are outlined in Table 1.

**Table 1:** Study characteristics (N=13).

First author	Year of publication	Country	Study design	Outcomes of interest
Arends et al [11]	2013	The Netherlands	Observational validation study	Physical activity
Bayraktar et al [39]	2021	Turkey	Cross-sectional study	Physical activity
Carbo et al [40]	2021	The Netherlands	Part of a prospective, longitudinal, observational cohort study	Physical activity
Coulter et al [30]	2019	United Kingdom	Prospective cohort study	Physical activity and sedentary behavior
Gossec et al [20]	2019	France	Prospective, multicenter, longitudinal, observational study	Physical activity and sedentary behavior
Jacquemin et al [29]	2017	France	Prospective, multicenter, longitudinal, observational study	Physical activity
Jacquemin et al [31]	2018	France	Prospective, multicenter, longitudinal, observational study	Physical activity
O’Dwyer et al [27]	2015	Ireland	Cross-sectional controlled study	Physical activity and sedentary behavior
Plasqui et al [28]	2012	The Netherlands	Observational case-control study	Physical activity
Swinnen et al [21]	2014	Belgium	Observational cross-sectional controlled study	Physical activity and sedentary behavior
van Genderen et al [41]	2015	The Netherlands	Multicenter cross-sectional study	Physical activity
van Genderen et al [42]	2014	The Netherlands	Cross-sectional case-control study	Physical activity
Yuksel et al [43]	2021	Turkey	Observational cross-sectional controlled study	Physical activity

Most studies were conducted in Europe (11/13, 85%), namely, the Netherlands (5/13, 38%) [11,28,40-42], France (3/13, 23%) [20,29,31], the United Kingdom (1/13, 8%) [30], Ireland (1/13, 8%) [27], and Belgium (1/13, 8%) [21]. Approximately 15% (2/13) of studies were conducted in Turkey [39,43]. Designs of the 13 studies included 1 (8%) observational validation study [11], 1 (8%) reproducibility study [40], 4 (31%) longitudinal studies [20,29-31], and 7 (54%) cross-sectional studies [21,27,28,39,41-43]. None of the studies reported interventions for the levels of physical activity or lifestyle. All studies (13/13, 100%) [11,20,21,27-31,39-43] focused on assessing physical activity and reported at least one corresponding outcome, whereas some studies (4/13, 31%) also reported outcomes related to sedentary behavior in patients with axSpA [20,21,27,30].

### Sample Characteristics

Table 2 presents participants’ descriptive characteristics. The 13 studies included covered a total of 819 patients with axSpA, of whom 490 (59.8%) were male. The mean sample size was 63 (SD 33.1), ranging from 24 [42] to 135 [41] participants with axSpA. The mean patient age was 44.43 (SD 4.4) years. Disease diagnoses were based on the Assessment of Spondyloarthritis international Society recommendations (6/13, 46%) [20,29-31,39,40], the modified New York criteria (4/13, 31%) [27,28,41,42], or both (2/13, 15%) [11,40]. Approximately 8% (1/13) of studies used the European Spondyloarthropathy Study Group recommendations for disease diagnosis [21]. Mean or median disease duration ranged from 4 [39] to 20.5 years [42].

All the studies used the Bath Ankylosing Spondylitis Disease Activity Index (13/13, 100%) [11,20,21,27-31,39-43], with mean or median scores ranging from 3.1 [20] to 4.5 [30]. Approximately 85% (11/13) studies used the Bath Ankylosing Spondylitis Functional Index [11,21,27,28,30,31,39-43], with mean or median scores ranging from 1.7 [31] to 4.4 [30]. Approximately 31% (4/13) of studies used the Bath Ankylosing Spondylitis Metrology Index [21,30,39,43], with mean scores ranging from 1.8 [39,43] to 3.05 [21].

Approximately 54% (7/13) studies included a healthy control group [21,27,28,31,41-43], including 281 healthy participants, of whom 169 (60.1%) were male. The mean sample size was 40.14 (SD 27.2), ranging from 24 [42] to 99 [41] control participants. The mean participant age across the healthy control groups was 42.56 (SD 16.8) years.

**Table 2:** Characteristics of patients with axSpA^a^.

First author			Participants, N		Males, n (%)		Age (years), mean (SD) or median (IQR)^b^		BMI, mean (SD) or median (IQR)^b^		Duration (years), mean (SD) or median (IQR)^b^		Criteria for diagnosis		BASFI^c^ score, mean (SD) or median (IQR)^b^		BASDAI^d^ score, mean (SD) or median (IQR)^b^		BASMI^e^ score, mean (SD) or median (IQR)^b^
Arends et al [11]			115		71 (62)		44.6 (12.1)		26.4 (4.4)		10.0 (0-42)^b^		ASAS^f^+NY^g^		3.8 (2.4)		3.7 (0-9)^b^		—^h^
Bayraktar et al [39]			58		32 (55)		39.0 (30.0-46.0)^b^		26.1 (23.7-28.7)^b^		4 (3-10)		ASAS		2.2 (0.5-3.8)^b^		2.8 (1.4-4.7)^b^		1.8 (1.1-2.95)^b^
Carbo et al [40]			45		23 (51)		50.7 (11.6)		—		27.0 (18-36)^b^		ASAS+NY		3.3 (1.4-5.7)^b^		3.4 (2.0-5.7)^b^		—
Coulter et al [30]			45		23 (46)		49.0 (11.7)		27.4 (5.6)		15.6 (11.2)		ASAS		4.4 (2.6)		4.5 (2.3)		3.6 (1.8)
Gossec et al [20]			73		41 (56.2)		41.2 (10.3)		24.6 (4.6)		10.8 (9.1)		ASAS		—		3.1 (2.0)		—
Jacquemin et al [29]			79		44 (55.7)		41.4 (10.2)		25.0 (4.6)		10.4 (8.9)		ASAS		—		3.3 (2.1)		—
Jacquemin et al [31]			74		43 (58.1)		41.3 (10.4)		25.3 (4.6)		10.4 (9.1)		ASAS		1.7 (1.8)		3.2 (2.1)		—
O’Dwyer et al [27]			39		32 (82.1)		40.0 (9.0)		28.6 (6.8)		6.0 (10.0)		NY		2.9 (3.8)		3.6 (2.2)		—
Plasqui et al [28]			25		15 (60)		48.0 (11.0)		26.2 (5.0)		19.0 (12.0)		NY		4.0 (2.2)		4.3 (2.2)		—
Swinnen et al [21]			40		24 (60)		44.38 (11.3)		26.3 (5.1)		11.4 (9.5)		ESSG^i^		3.52 (2.5)		3.7 (2.6)		3.1 (1.2)
van Genderen et al [42]			24		14 (58.3)		48.0 (11.0)		26.0 (4.6)^b^		20.5 (22.0)^b^		NY		3.8 (2.1)^b^		4.0 (3.7)^b^		—
van Genderen et al [41]			135		81 (80)		51.0 (13.0)		26 (4.3)		16.5 (12.1)		NY		4.1 (2.6)		4.3 (2.2)		—
**Yuksel et al [43]**																			
	AS^j^	34		47 (70.1)		41.0 (31-46)^b^		26.1 (22.9-29.6)^b^		8.0 (4-13)^b^		ASAS		2.4 (0.5-3.9)^b^		3.4 (1.5-5.8)^b^		2.1 (1.5-3.9)^b^	
	Nr-SpA^k^	33		47 (70.1)		37.0 (32-40)^e^		26.3 (25.4-28.7)^e^		4.0 (2-9)^e^		ASAS		1.2 (0.6-2.9)^e^		2.4 (1.4-5.4)^e^		1.5 (1.1-2.0)^e^	

^a^axSpA: axial spondyloarthritis.

^b^Outcomes are reported with median (IQR) values.

^c^BASFI: Bath Ankylosing Spondylitis Functional Index.

^d^BASDAI: Bath Ankylosing Spondylitis Disease Activity Index.

^e^BASMI: Bath Ankylosing Spondylitis Metrology Index.

^f^ASAS: Assessment of Spondyloarthritis international Society.

^g^NY: modified New York criteria.

^h^Not available

^i^ESSG: European Spondyloarthropathy Study Group.

^j^AS: ankylosing spondylitis.

^k^nr-SpA: nonradiologic form of axial spondyloarthritis.

### Methods of Measuring Physical Activity and Sedentary Behavior

Table 3 presents the methods used to objectively assess physical activity or sedentary behavior among patients with axSpA. Information regarding wearable devices (eg, device name, manufacturer, and sensor), assessment methods (device location, length of monitoring, requisite conditions for valid monitoring, visualization of physical activity by the participants, and instructions to the participants on physical activity), and outcomes (physical activity and sedentary behavior) are reported.

**Table 3:** Wearable device and monitoring characteristics.

First author	Device name	Manufacturer (country)	Sensor	Device location	Length of monitoring	Requisite conditions for valid monitoring	Visualization of physical activity by the participants	Instructions to the participants on physical activity	Outcomes reported
Arends et al [11]	The ActiGraph: GT1M	ActiGraph (United States)	Uniaxial accelerometer	Right hip	7 consecutive days	Minimum wear time of 10 hours per day and 5 days with both weekend day	No information	No information	Kilocounts per day and mean wear time
Bayraktar et al [39]	GT3X	ActiGraph (United States)	Triaxial accelerometer	Waist	7 consecutive days	Patients not wearing accelerometer as instructed (<10 hours per day wear; <7 days total wear) were removed from the analysis	No information	No information	Total activity duration and activity duration intensity (light or moderate or vigorous); METs^a^ for total and for each physical activity intensities
Carbo et al [40]	GT3X	ActiGraph (United States)	Triaxial accelerometer	Right hip	7 consecutive days	Data excluded if accelerometer worn <10 hours per day, for <5 days or for <2 weekend days	No information	No information	Total activity kilocounts and activity duration intensity (light, moderate, and vigorous) in minutes per week
Coulter et al [30]	The activPAL3	PAL Technologies Ltd (Scotland)	Triaxial accelerometer	Anterior thigh of the dominant leg	7 consecutive days	Minimum wear time of 24 hours for a valid day	No information	No information	Daily standing, walking, sedentary time, and steps per day
Gossec et al [20]	Withings Activité Pop	Withings (France)	—^b^	Wrist	90 consecutive days	—	“patients could visualize their physical activity on their smartphones.”	“No instruction about physical activity was given to the participants”	Steps per minute
Jacquemin et a. [29]	Withing Activité Pop	Withings (France)	—	Wrist	90 consecutive days	—	“patients could visualize their physical activity on their smartphones.”	“No instruction about physical activity was given to the participants”	Steps per day, total activity duration, and activity duration in moderate to vigorous intensity
Jacquemin et al [31]	Withing Activité Pop	Withings (France)	—	Wrist	90 consecutive days	—	No information	“No intervention was specifically performed to increase physical activity, and no instruction about physical activity was given to the participants.”	Steps per day, morning step count, total activity duration, and activity duration in moderate to vigorous intensity
O’Dwyer et al [27]	RT3	Stayhealthy Inc (United States)	Triaxial accelerometer	Right hip	7 consecutive days	Minimum wear time of 10 hours per day, including at least one weekend day	No information	No information	Counts per day
Plasqui et al [28]	Tracmor	Philips Research (the Netherlands)	3 uniaxial piezoelectric accelerometers	Lower back	7 consecutive days	—	No information	No information	Kilocounts per day
Swinnen et al [21]	SenseWear Pro 3 Armband	Bodymedia Inc (United States)	Biaxial accelerometer	Right triceps muscle	5 consecutive days	Minimum wear time of 1296 minutes, corresponding to 90% of a 24-hour period, including both weekend days	No information	No information	Weekly average of kilocounts per day
van Genderen et al [42]	Tracmor	Philips Research (the Netherlands)	3 uniaxial piezoelectric accelerometers	Lower back	7 consecutive days	Minimum wear time of 10 hours for a valid day	No information	No information	Kilocounts per day and mean wear time
van Genderen et al [41]	GT3X	ActiGraph (United States)	Triaxial accelerometer	Lower back	7 consecutive days	Minimum wear time of 10 hours for a valid day	No information	No information	Vector magnitude counts, counts per day, and counts per minute
Yuksel et al [43]	GT3X	ActiGraph (United States)	Triaxial accelerometer	Waist	7 consecutive days	Not specified; however, participants “instructed to wear the device for at least 10 h/day except for water-related activities such as showering or swimming”	No information	No information	Activity duration (light, moderate, vigorous) in minutes per week

^a^MET: metabolic equivalent of task.

^b^Not available

All studies (13/13, 100%) [11,20,21,27-31,39-43] reported wearable device–measured physical activity outcomes, whereas some (4/13, 40%) studies reported wearable device–measured sedentary behavior outcomes among patients with axSpA [20,21,27,30]. Only 15% (2/13) of studies provided information on the visualization of physical activity levels by the participants [20,29], and 23% (3/13) of studies provided instructions to the participants on physical activity [20,29,31].

### Types of Sensors

All 13 studies used accelerometer-based wearable devices, with 6 (46%) using triaxial accelerometers [27,30,39-41,43], 2 (15%) using 3 uniaxial piezoelectric accelerometers [28,42], 1 (8%) using a biaxial accelerometer [21], and 1 (8%) using a uniaxial accelerometer [11]. Approximately 23% (3/13) of studies did not mention the type of accelerometer [20,29,31]. The brands used were ActiGraph (5/13, 38%) [11,39-41,43], Withings (3/13, 23%) [20,29,31], Philips Research (2/13, 15%) [28,42], PAL Technologies (1/13, 8%) [30], Stayhealthy (1/13, 8%) [27], and Bodymedia (1/13, 8%) [21].

The Withings device was the Withing Activité Pop, an accelerometer-based activity tracker worn on the wrist. ActiGraph devices included the GT1M, a uniaxial accelerometer fixed on the participant’s right hip, and the GT3X, a triaxial accelerometer placed on the lower back using a belt. The Tracmor sensor is a combination of 3 uniaxial piezoelectric accelerometers that are fixed to the lower back. PAL Technologies’ activPAL3 is a triaxial accelerometer fixed to the anterior thigh of a participant’s dominant leg. Stayhealthy’s RT3 is a triaxial accelerometer worn on the right hip. Bodymedia’s SenseWear Pro 3 Armband is a biaxial accelerometer worn on the back of the right triceps muscle.

Wearable devices were secured on the wrist (3/13, 23%) [20,29,31], lower back (3/13, 23%) [28,41,42], right hip (3/13, 23%) [11,27,40], waist (2/13, 15%) [39,43], anterior thigh of the dominant leg (1/13, 8%) [30], and right arm (1/13, 8%) [21].

### Monitoring Protocol

Approximately 69% (9/13) of studies used 1-week monitoring (7 consecutive days) [11,27,28,30,39-43], and 8% (1/13) used a 5-day period (including both weekend days) [21]. Approximately 23% (3/13) of studies used 3-month monitoring and follow-up [20,29,31]. None of the studies assessed only days of the week. Monitoring was considered complete when wearable devices were worn on both weekend days in 38% (5/13) of studies [11,21,39,40,43] or when 1 weekend day was included in the follow-up period in 8% (1/13) of studies [27]. Approximately 54% (7/13) of studies imposed wearing the tracker for at least 10 hours per day [11,27,39-43], 10% (1/13) imposed wearing trackers for at least 1296 minutes (corresponding to 90% of 24 hours) [21], and 10% (1/13) imposed a minimum wear time of 24 hours per day [30]. Approximately 23% (3/13) of studies reported wear time [11,40,42] and 23% (3/13) others reported activity duration or time spent on specific activities (walking and standing) [20,29,31].

### Physical Activity Outcomes

Twelve objective measures of physical activity were used to assess patients with axSpA in the 13 studies: 4 (31%) studies reported steps per day [20,29-31], 6 (46%) reported activity counts [11,27,28,40-42], 4 (46%) reported total activity duration [29,31,39,41], 2 (15%) reported energy expenditure [21,39], 2 (15%) reported levels of physical activity [21,28], and 4 (31%) reported average wear time in hours per day [11,21,40,42]. Approximately 69% (9/13) of studies also reported activity intensity [21,27,29-31,39-41,43] using six different expressions of measurement: light, moderate, vigorous, and very vigorous levels of activity levels [21,27,39-41,43]; minutes spent in moderate to vigorous activity [21,27,29-31]; and mean walking-event cadence [30].

The number of steps per day was reported as the average daily step count [20,29-31]. Activity counts were reported as the average of daily activity counts [11,27,28,41,42]. Total activity duration was reported as the sum of minutes involving at least 20 steps recorded in a week [29] or as active minutes in a day, derived from either step count (minutes with at least 20 steps recorded) [31] or activity count [41]. Energy expenditure was expressed as the metabolic equivalent of task (MET) hours per day [21] or MET minutes per week [39]. METs were used to report the overall and objective levels of physical activity in 15% (2/13) of studies [21,28]. Activity intensity was obtained from activity counts [21,27,30,41] or number of steps [29,31]. Established cutoff points were used to convert raw data from daily activity counts [27,41] or MET values [21,30] into each activity intensity. The average time spent doing light, moderate, and vigorous activities was reported in minutes per day [21,27,41] or hours per day [27]. One of the studies reported the score for very vigorous activity and expressed it in minutes per day [21]. The time spent performing light to vigorous activities was expressed in minutes per day [21,27,31] or minutes per week [29,30,39,40,43]. Another study reported cadence using steps per minute [30].

### Sedentary Behavior Outcomes

A total of 5 measures were used to assess sedentary behavior in patients with axSpA. These included (1) the number of sitting events (1/13, 8%) [30], (2) total sitting time (1/13, 8%) [30], (3) the number of bouts of prolonged sitting time (>30 minutes; 1/13, 8%) [30], (4) the total duration of this prolonged sitting time (1/13, 8%) [30], and (5) duration of sedentary behavior (3/13, 23%) [21,27,41].

Cutoff values based on daily activity counts [27,41] or MET values [21,30] were used to derive sedentary time from the raw data. Coulter et al [30] also reported sitting events per day, duration of sitting time in hours per day, and the number of periods of prolonged sitting time (>30 minutes).

## Discussion

To the best of our knowledge, this is the first systematic review to identify and synthesize available evidence on the use of wearable activity trackers as outcome measures for physical activity and sedentary behavior in patients with axSpA. For the sake of clarity, we discuss our findings through three main themes: (1) the wearable devices themselves, (2) reference outcomes for physical activity and sedentary behavior assessment using wearable devices, and (3) monitoring protocols and assessment methods.

### Wearable Devices

Our findings showed the broad use of wearable devices, mostly incorporating triaxial accelerometers [27,28,30,39-43], and less use of simple devices such as pedometers [20,29,31] or uniaxial or biaxial accelerometers [11,21]. Among the studies included in our synthesis of directly comparable data (Table 2), accelerometers were the most frequently used direct measuring devices.

To implement these wearables in clinical practice, measurements should be both feasible (ie, used by patients and health professionals) and accurate (ie, validity and reliability) [44-46].

Monitoring of health and physical activity seems feasible in patients with axSpA. Indeed, in a recent study by Jacquemin et al [31], 157 patients reported that wearing a wristwatch-type device for 3 months was acceptable, with a mean acceptability score of 8 out of 10 [31]. However, the interpretation of the data provided by these devices requires digital health skills that not every patient with axSpA may have. The implementation of wearables in clinical practice also necessitates the formation and training of health professionals supporting patients with the use of wearable activity trackers. None of the included studies have addressed this issue.

Depending on their purpose (ie, with specific conditions of use), the validity of activity trackers can vary significantly, making them more or less suitable for research purposes. Some activity trackers are specifically designed for research purposes (*research activity trackers*), with relatively short-term use, fewer needs for the interface with the users, and more precise and detailed parameters. This is the case for the ActiGraph [47,48], Philips Research [48], and ActivPAL [48,49] sensors, which have been widely validated against doubly labeled water in healthy control populations and presented a high degree of accuracy [47-49]. Other activity trackers (such as Fitbit or Withings devices) are primarily designed for consumers to monitor and improve their physical activity levels, are easy to wear, and are adapted for long-term use. At this point, it is important to note that previous studies have reported that the validity of these devices is lower than that of research activity trackers [50-53], the estimation of energy expenditure was outside the acceptable accuracy [54-57], and the availability of raw data is not always warranted.

Interestingly, we found no published studies assessing the psychometric properties of wearable devices to monitor physical activity and sedentary behavior in our specific population, neither in free-living conditions nor in a more standardized environment, such as a laboratory. Therefore, it would be appropriate to conduct studies addressing the metrological properties of these devices in this population. Importantly, patients with axSpA seem to present with motor and gait specificities [36,58] that could affect the validity of wearables designed to monitor walking activity.

The positioning of the wearable devices on the body should also be considered. The 3 main locations used were the wrist [20,29,31], lower back [28,41,42], and hip [11,27,40]. In the general population, previous studies have reported that where devices are placed on the body has a significant impact on the accuracy of the number of steps counted during various walking activities [44,46,59,60]. The major trend reported in these studies was the outperformance of hip-worn devices. For example, in the laboratory-based validation protocol described by Kooiman et al [46], a hip-worn activity tracker (Fitbit Zip, Fitbit Inc) had the highest validity and reliability in counting healthy participants’ steps. Hip-mounted devices were also the best for counting steps at the preferred walking speeds of healthy individuals, with a lower absolute mean relative error [60]. However, when walking speeds decreased, the wrist-worn devices in the same study provided more accurate step count estimations than the hip-worn devices [60].

Previous studies have shown that physical activity and sedentary behavior can be modified in patients with axSpA compared with healthy controls. Indeed, if no significant differences were found between patients with axSpA and healthy controls in light physical activity [21,27,41,43], counts per day [27,28,41,42], or duration of sedentary behavior [21,27,41], 31% (4/13) of studies found that patients with axSpA performed significantly less vigorous activity [21,27,41,43]. Results regarding durations of moderate or moderate to vigorous activity were inconsistent (ie, some studies found significantly less moderate physical activity [41,43] or moderate to vigorous activity [21] in patients with axSpA, whereas others did not [21,27]).

Moreover, considering the symptoms caused by spondyloarthritis, such as limitations in the sagittal range of motion of the lower limbs during gait [61,62] and lower gait speed [63], previous results in the literature regarding wearable devices in axSpA may be questionable. Future studies on how the location of wearable devices affects the overall accuracy of measurements of physical activity and sedentary behavior among this specific population are needed, particularly regarding the gait specificities of patients with axSpA [36,58].

Furthermore, only 15% (2/13) of studies mentioned that participants could visualize physical activity [20,29], and 23% (3/13) of studies mentioned that they provided no instructions on physical activity [20,29,31]. Wearing a wearable tracker and visualizing physical activity can increase the physical activity of participants [64]. Thus, future studies on wearables and physical activity should include information on visualization and instructions on physical activity.

### Reference Outcomes for Physical Activity and Sedentary Behavior Assessment Using Wearable Devices

The studies included in this systematic review reported two main categories of outcomes related to physical activity: first, the number of daily steps taken [20,29-31], and second, data based on the daily activity counts recorded by accelerometers [11,21,27,28,39-43]. These 2 types of data allowed us to estimate the time spent doing activities of different intensities (ie, light, moderate, or vigorous). In other words, using threshold values, it is possible to estimate the intensity of an activity based on the number of steps or activity counts.

In contrast, even if recording the number of steps per day (4/13, 31% studies) [20,29-31] requires fewer raw data than recording the activity count, parameters related to the activity count were used slightly more for tracking individual behavior (9/13, 69% studies) [21,27,29-31,39-41,43]. Indeed, monitoring techniques based on activity count data require devices with greater memory and storage capacity; however, recent technological advances have made data compression and storage problems almost irrelevant. There is extensive literature related to activity count cutoff points [65-71] but not to step cutoff points, which could explain why activity counts were more common in the present review. Further investigation in a laboratory-type setting is needed to draw firm conclusions on the pros and cons of each measurement method in the axSpA population. It would also be appropriate to determine specific cutoff points for this population for both step and activity counts.

Using thresholds allowed us to categorize low, moderate, vigorous, and very vigorous activities. However, in line with the recommended levels of physical activity already stated for the general population [72], and especially in this population [73], we believe that it would be preferable to group individuals’ moderate-intensity and vigorous-intensity activities into one moderate to vigorous activity category [74], which would also facilitate future comparisons with the rest of the literature.

Another way of expressing physical activity levels is by using METs. METs are a method of expressing an activity’s energy costs, and they refer to energy expenditure rather than an activity’s intensity. Using METs would seem to be more relevant and more likely to accurately report an individual’s true level of physical activity or energy expenditure [75]. Nevertheless, the lack of available data did not allow us to validate this outcome’s use among pathological populations; therefore, using kilocalories was a preferable way of expressing energy expenditure. An increasing number of studies used activity counts to report physical activity levels in the axSpA patient population [11,27,28,41,42]; however, it would seem advisable to assess this outcome under standardized laboratory and open-field conditions.

The number of daily steps remains the outcome of choice for monitoring an individual’s activity level [44,76-78]. This enables the particularly straightforward detection of decreases in ambulatory activities that prevent the onset of runaway evolution in the disease or marked disease flares [29]. As one of the studies already addressed this question by linking daily numbers of steps over 3 months to acute disease flare-ups [29], we believe that data over longer periods could also be of interest. Just as Tudor-Locke et al [79] reported changes in daily activity patterns over the course of a year, we think it would be interesting to track the daily number of steps taken by patients with axSpA over 1 year.

The literature concerning assessments of sedentary behavior is much more scattered, and more studies are needed to confirm the trends reported to date. As with the intensity of physical activity, sedentary behavior can be defined using certain thresholds. Some researchers have used activity count [27,41,42], whereas others have estimated sedentary behavior using METs provided by the manufacturers’ algorithms [30].

At present, only one method of monitoring sedentary behavior exists for patients with axSpA, although it has different outcomes. This method uses wearable trackers and the acceleration data obtained from them. The outcomes are overall reports of sedentary time based on thresholds and detailed reports of sitting times and the number of sitting events.

To increase the monitoring precision and for comparative purposes, we suggest that all studies clearly mention the duration of carrying the wearable devices.

### Monitoring Protocols and Assessment Methods

The studies included in this systematic review reported two ways of monitoring sedentary behavior and physical activity levels of patients with axSpA. We found studies with short follow-up periods of 5 to 7 days [11,21,27,28,30,39-43] and others with follow-up periods of >90 days [20,29,31], depending on each study’s objectives. Importantly, sufficient daily wear time and a sufficient number of follow-up days, including specific weekend days, had to be ensured for that follow-up to be valid. Interestingly, all studies included weekends in their monitoring. For example, Gossec et al [20] identified the critical time intervals for classifying activities and tracking accuracy. These authors also reported that the significantly different activities performed on Saturday afternoons were associated with the detection of axSpA flare-ups and changes in flare-up state [20]. When examining the general population data [79], the days of the week included in the follow-up may have influenced the monitoring results. Furthermore, owing to the technical properties of wearable devices, some activities, such as swimming, could not be monitored.

The length of monitoring (ie, 1 week or several weeks) and days included in the monitoring (ie, weekday, workday, day off, and weekend) should be harmonized as they influence physical activity performance [20,80,81]. If reliable results can often be found with 1 week of monitoring, longer monitoring can help health professionals capture days of the week in which the participant is always inactive to further adapt the intervention program [80].

### Limitations and Perspectives of the Review

Wearable trackers are promising as they have the potential to better monitor the physical activity and sedentary behavior evolution of patients with axSpA and study its impact on the disease. Mobile health, including trackers, permits health monitoring when outside health structures and could limit the number of visits to the hospital or clinic [82]. The literature on wearable trackers is rapidly building, and it is possible that some studies were published between the search and publication of this review. Moreover, most systematic reviews were limited by the small number of studies included. To avoid this, the search strategy included all fields and was not limited to the titles and abstracts. We used a thorough systematic and transparent methodology to conduct this review [32]. Despite this, only a few studies were included in the review, and we encountered some challenges when comparing across studies because of varying methods and reported results.

Furthermore, although the present review did not focus on the role of wearables as interventions to improve physical activity and sedentary behavior, this area of research could represent a relevant future research direction. Indeed, trackers can also be used as an intervention to motivate users to increase physical activity and decrease sedentary behavior [17,64,83-85] and could further prevent inactivity- or sedentary-related diseases (eg, cardiovascular diseases) [12].

### Conclusions

This review identified and synthesized currently available evidence on the use of wearable activity trackers as outcome measures for physical activity and sedentary behavior in patients with axSpA.

We have underlined some trends regarding (1) the types of wearable devices used, (2) the outcomes reported, and (3) the follow-up protocols used. To date, few studies have used wearable devices to quantify physical activity among patients with axSpA, and the methods used have been heterogeneous. To fill this gap in knowledge and the literature, we suggest that future research focus on testing the feasibility and accuracy of physical activity and sedentary behavior assessments in patients with axSpA. The best locations to position the sensors should also be considered. This should occur in both the short-term, controlled, and supervised conditions of a laboratory environment and the long-term, varied, and everyday conditions of normal living environments.

## Abbreviations

axSpA: axial spondyloarthritis
MET: metabolic equivalents task
PRISMA: Preferred Reporting Items for Systematic Reviews and Meta-Analyses
PROSPERO: International Prospective Register of Systematic Reviews
